# Progress in Fevers

**Published:** 1901-09-14

**Authors:** 


### PROGRESS IN FEVERS.

(*Continued from page* 317.)

**Typhoid Fever.**—H. H. Tooth [10] relates his experience of enteric fever in the Orange River Colony. The outbreak began among the Modder River force before the relief of Kimberley or the victory at Paardeberg. Water, sandstorms, and flies have the credit of disseminating the disease. Flies were a great pest, and showed a special predilection for typhoid patients. (Sir W. Church [11] found it possible on entering a hospital tent to identify typhoid cases by the number of flies congregated around them.) The first frosts coincided with a rapid decline in the epidemic and a great mortality among the flies. Sandstorms were very frequent, and sand, almost inevitably contaminated by fæcal and urinary accumulations, entered largely into every article of food. Personal infection from man to man must also have played a considerable part, for the men slept 15 in a tent, water, even for drinking purposes, was short, and many men kept on duty during the insidious onset of the disease. Antecedent disease, gastro-enteritis, diarrhœa and dysentery probably figured largely in determining typhoid infection. Typhoid frequently followed dysentery, and diarrhœa was almost universal. Horton-Smith is quoted as having recently shown experimentally how the virulence of the typhoid bacillus is increased by the presence in the blood of other toxins such as that of bacillus coli. Probably most of the army received typhoid bacilli at one time or another into the alimentary canal, and the virulence of these bacilli is likely to have become increased by an attack of simple gastro-enteritis, with consequent multiplication of bacilli normally present in the intestine in small quantities. In treatment diet was regulated by the digesting power of the stomach—finely minced meat and bread and butter not being regarded as harmful in cases of good digestion. Alcohol was used very sparingly, and only in the severer cases, never more than 6 ozs. in 24 hours. Perforation is marked by a rise in pulse and respiration rates and sudden fluctuation of temperature. Acute local pain, abolition of liver dulness, and thoracic breathing may be little marked or even absent.

Sir W. Broadbent [12] regards it as proved that diarrhœa and dysentery favour the onset of typhoid. He considers also that influenza increases susceptibility. Possibly there are varieties of the bacilli which may account for differences in immunity and in the character of the fever. He would prefer preventive inoculation with a toxin obtained from cases where the epidemic was raging. Rise of pulse and respiration are important as indications of perforation; breathing is always thoracic unless the symptoms are masked by opium.

A. Crombie [13] points out as characteristic of the South African epidemic the frequency of second attacks, of thrombosis, and of subsequent muco-colitis. The extraordinary richness of the rash was also remarkable. A consideration of the clinical symptoms suggested a mixed infection. D. Melville,[14] on the other hand, found as a result of analysing 295 cases of enteric treated by him in Ladysmith, that rash was absent in 40 per cent., while in 20 per cent. it was thickly scattered over the trunk and extremities, and followed the usual distribution in the remainder of the cases. In the disease as seen in Cape

Colony the rash is more frequently absent than present. There seems no relation between the rash and the character of the attack. Diarrhœa among his cases was exceptional, constipation was very prevalent and refractory—it yielded best to turpentine enemas. Pneumonia, a common cause of death in the country, became very frequent in June. His cases were all kept on fluid diet, and 2 minims of carbolic acid were given every four hours as routine treatment. J. W. Washbourn[15] also found constipation very common among the cases treated in the Imperial Yeomanry Hospital, Pretoria. Diarrhœa and hæmorrhage were uncommon, but phlebitis occurred with great frequency. Death frequently occurred in otherwise mild cases from cardiac failure in patients much over-fatigued, or who had been carried long distances. A good many cases were noticed in which short attacks of pyrexia preceded the onset of enteric fever, the temperature falling to normal for a few days between. A good many cases also of undetermined fever were observed, in which irregular pyrexia was accompanied by malaise but no definite symptoms. These cases showed considerable resemblance to Malta fever, and in some cases the blood agglutinated the Malta fever coccus in dilutions of 1 in 80, whilst no reaction was obtained with typhoid cultures.

Horton Smith[16] considers it even more important to disinfect the urine than the fæces in typhoid patients. He advocates disinfection in the bladder by the use of 10 grains of urotropin thrice daily. Occasional dysuria was the only bad symptom noticed during weeks of administration. Neufeld[17] also draws attention to the danger of typhoid infection arising from the immense number of bacilli passed in the urine. The urine should receive as careful disinfection as the stools. He quotes one reported case where large numbers of bacilli were found in the urine of a patient treated in hospital five years previously for typhoid fever. The urine had been turbid ever since. In all cases of typhoid he recommends the administration of urotropin from the commencement of the third week of the disease, to be continued until the urine is perfectly clear to the naked eye, showing no trace of turbidity. Gwyn[18] also insists on the necessity of disinfecting the urine. For disinfection before excretion, he, too, considers urotropin the only effective internal drug. Irrigation of the bladder with solutions of mercuric chloride in strengths of 1 in 100,000, or 1 in 50,000 are also very useful.

Osler[19] considers perforation most likely to occur in cases showing diarrhœa and tympanites. Among other signs to be noted, such as the condition of the abdomen, the character and situation of the pain and changes in the temperature and pulse rate, sudden increase in the respirations and shallow or sighing respiration are to be looked for. Watch also should be kept on the leucocyte count. Finney[20] considers most stress in diagnosing perforation is to be laid on sudden continuous abdominal pain, nausea, vomiting, and leucocytosis. Cushing and Thayer[21] find the last-named is often absent. T. McCrae[22] publishes some observations made on typhoid patients in Professor Osler's wards, noting the incidence of abdominal pain. In all cases of perforation it was severe, and in most cases sudden in onset. Generally it passed off quickly and became paroxysmal. Occasionally pain was present some days before perforation occurred. C. Russell[23] considers leucocytosis sometimes of value in determining the occurrence of perforation. Leucocytosis is the rule, although there are many exceptions. Its presence may justify operation in a doubtful case. Foord Caiger[24] discussing the differential diagnosis of enteric, points out that there are five symptoms which together are positive indications of the nature of the fever, viz., the rash—papules confined to the trunk, present in 70 per cent. of all cases and almost always present in adults, enlarged spleen, Widal's reaction, bronchial catarrh, which is very frequent in the early stages, and deafness which is very common in the first week and is due generally to a simple Eustachian catarrh. With regard to prognosis mortality rises with age. Fat muscular people and those disposed to renal disease or gout are bad subjects. Intemperance, prolonged mental strain, and over-fatigue are very unfavourable. So are tympanites and violent diarrhœa, together with high temperature, abdominal pain and pronounced nervous symptoms, while lung complications, hæmorrhage and perforation are of grave import.

V. Dickenson[25] deals with two cases of typhoid complicated by rigors and sudden rises of temperature not of the nature of ordinary relapses. The temperature occasionally rose to 105° or 106° Fahr., the initial rigor was severe, but the fever was brief and the constitutional symptoms almost nil. He points out that Gee considers such febrile attacks due to constipation, and that rigors during or following typhoid are not necessarily of grave significance. Abercrombie is also quoted as having observed the association with constipation. Carslaw[26] reports two cases of paralysis following typhoid. In one, a soldier who suffered from enteric in South Africa, extensive asymmetrical paralysis of the muscles of both upper limbs has resulted. In the second case, a girl of 22, who subsequently developed valvular disease of the heart, hemiplegia occurred during the illness.

Barjon and Lesieur[27] record a case of typhoid in a boy of 17 who had previously had typhoid fever, and later, acute rheumatism. Fever and rose spots were present, a blood cultivation showed Eberth's bacillus in abundance, and Widal's reaction was obtained several times. Splenic enlargement and intestinal symptons were absent, but diarrhœa occurred later. There was marked arthritis in the feet and hands. *Post mortem* no intestinal lesions except congestion of the colon were found. Granulations on the mitral and aortic cusps, pulmonary œdema, and fatty liver were present. These observers consider that the first attack of typhoid rendered the spleen and intestine immune, and that the brunt of the second attack fell on the joints already damaged by a previous attack of rheumatism. Schottmüller[28] records a case of fever clinically resembling typhoid in which continued fever, enlarged spleen, bronchitis, and roseola were present. The stools were not typical. Bacilli were found in the blood which morphologically and in many other respects resembled typhoid bacilli, but Widal's reaction was not present, and the bacilli did not form indol. Eshner and Weisenberg[29] record two cases of hæmorrhagic typhoid. Both patients were addicted to alcoholic excess. In one the hæmorrhages were practically universal, and the other confined to the skin. The suggestion is made that possibly such cases as these which occur also in other fevers, are examples of a hæmorrhagic purpura occurring in the course of the primary disease. Bourdillon[30] describes a case of colotyphus, a case of typhoid fever in which the lesion affected the large intestine. These cases are difficult to distinguish from the ordinary ones in which some accessory ulceration of the colon occurs. Abundant, profuse, and very fœtid diarrhœa is present, bright blood may appear, and asthenia is marked. Baccarani[31] has seen unusual symptoms, such as pulsus paradoxus, cough on handling the enlarged spleen, and desquamation of the thorax and abdomen. Kinnicutt[32] has seen two cases of orchitis and epididymitis occurring in the course of typhoid. Remlinger[33] finds that no constant relation exists between the state of the deep reflexes and the severity of the disease, but in severe adynamic forms the reflexes tend to be exaggerated.

A. Nadeau[34] warmly advocates the revival of the use of calomel in the treatment of typhoid. He administers it after the German method as a 10-grain dose given at night in gelatine capsules or wafers for three successive nights, and followed next morning by a saline laxative. It must not be given after the first week. With this treatment the disease becomes "a slight indisposition lasting two or three weeks." Owing to its threefold action as germicide, purgative, and chologogue, all bad symptoms are abated. Other therapeutic measures are employed later, such as β, or better, benzo-naphthol and ice packs or ice enemata, which are very useful for their general tonic effect. Gerest and Rigot[35] advocate the use of pyramidon, a derivative of antipyrin, as an antipyretic, and claim for it a quicker and more lasting action without adverse symptons. Jez and Kluk-Kluczycki[36] claim success from the use of a new "antityphoid extract," which consists of a solution of antitoxins extracted from rabbits previously immunised by injection of typhoid bacilli. The extract is a harmless reddish alkaline fluid which may be given by the mouth.

[10] Lancet, Mar. 16. [11] Brit. Med. Jour., Mar. 30. [12] Ibid. [13] Ibid. [14] Brit. Med. Jour., April 20. [15] Ibid. [16] Brit. Med. Jour., Mar. 30, p. 773. [17] Therapist, Mar. 15. [18] Amer. Jour. Med. Sc., May. [19] Ibid. [20] Jno. Hopkins Hosp. Reports, 1900. [21] Ibid. [22] Lancet, June 1. [23] Lancet, May 18. [24] Clin. Jour., April 17. [25] Edin. Med. Jour., April. [26] Glas. Med. Jour., Mar. [27] Brit. Med. Jour., May 11. [28] Amer. Jour. Med. Sc., April, p. 453. [29] Amer. Jour. Med. Sc., Mar. [30] Brit. Med. Jour., June 1. [31] Brit. Med. Jour., May 18. [32] Med. Rec., May 25. [33] Brit. Med. Jour. Mar. 23. [34] Merks Archives, Jan. [35] Les Nouveaux Remèdis, Feb. 24. [36] Brit. Med. Jour, Mar. 30.

